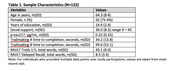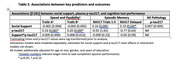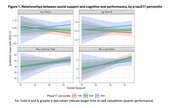# Exploring connectedness as a source of cognitive resilience in Black middle‐aged and older adults: Social support, plasma *p*‐tau217, and cognition

**DOI:** 10.1002/alz70860_107457

**Published:** 2025-12-23

**Authors:** Megan L. Zuelsdorff

**Affiliations:** ^1^ Wisconsin Alzheimer's Disease Research Center, University of Wisconsin‐Madison, SMPH, Madison, WI, USA; School of Nursing, University of Wisconsin‐ Madison, Madison, WI, USA; Wisconsin Alzheimer's Disease Research Center, University of Wisconsin School of Medicine and Public Health, Madison, WI, USA; Division of Geriatrics and Gerontology, Department of Medicine, University of Wisconsin School of Medicine & Public Health, Madison, WI, USA; University of Wisconsin ‐ Madison School of Nursing, Madison, WI, USA; University of Wisconsin School of Medicine and Public Health Alzheimer's Disease Research Center, Madison, WI, USA; University of Wisconsin School of Medicine and Public Health, Madison, WI, USA

## Abstract

**Background:**

Epidemiological evidence suggests that excess risk for all‐cause dementia in minoritized populations is preventable; inclusive prevention science will identify protective factors for communities facing highest risk. Social connectedness appears to reduce dementia risk: plausible mechanisms include (I) direct influence on neuropathology, or (II) indirect influence through cognitive reserve processes. In a richly characterized sample of Black middle‐aged and older adults, we explored associations between (i) social support, cognition, and plasma *p*‐tau217, a biomarker of Alzheimer's pathology; and (ii) social support as a source of resilience to pathological burden.

**Method:**

Participants (*N* = 122) were enrolled through the African Americans Fighting Alzheimer's in Midlife study into the Wisconsin Registry for Alzheimer's Prevention or Alzheimer's Disease Research Center studies. All were unimpaired and had complete neuropsychological, psychosocial, and plasma *p*‐tau217 data. Multivariable linear regression models assessed cross‐sectional predictor‐outcome relationships. The key predictor was perceived availability of social support, measured with the Medical Outcomes Study – Social Support survey. Outcomes of interest included continuous *p*‐tau217 level and performance on tests of (a) speed and flexibility and (b) immediate and delayed memory. Reserve mechanisms were assessed with a moderation approach, interacting support scores with plasma *p*‐tau217 values to predict cognition.

**Result:**

Sample characteristics are in Table 1. In adjusted models (Table 2), social support scores positively associated with delayed memory test performance but not speed and flexibility. Social support did not associate with *p*‐tau217 level. Further, there were no significant interactions between social support and *p*‐tau217; higher support scores predicted better delayed memory regardless of Alzheimer's pathology burden (Figure 1).

**Conclusion:**

Greater availability of a key resource, social support, predicted better episodic memory but not speed and flexibility within this small, Black cohort. In contrast, *p*‐tau217, a biomarker of AD pathology, was related to greater speed and flexibility but not episodic memory. Supportive social relationships may act on recall abilities independent of Alzheimer's‐specific pathology: social support did not associate with *p*‐tau217. Facilitating social connection may be a promising nonpharmacological intervention strategy in Black communities but future studies must examine the role for additional dementia pathologies, and in larger samples.